# ILC2s and Adipose Tissue Homeostasis: Progress to Date and the Road Ahead

**DOI:** 10.3389/fimmu.2022.876029

**Published:** 2022-06-16

**Authors:** Takuma Misawa, Marek Wagner, Shigeo Koyasu

**Affiliations:** ^1^ Laboratory for Immune Cell Systems, RIKEN Center for Integrative Medical Sciences, Yokohama, Japan; ^2^ Department of Biomedicine, University of Bergen, Bergen, Norway

**Keywords:** innate lymphoid cells, ILC2, adipose tissue, metabolism, cytokines, intestine

## Abstract

Group 2 innate lymphoid cells (ILC2s) were initially identified as a new type of lymphocytes that produce vigorous amounts of type 2 cytokines in adipose tissue. Subsequent studies revealed that ILC2s are present not only in adipose tissue but also in various other tissues such as lung and skin. ILC2s are generally recognized as tissue-resident immune cells that regulate tissue homeostasis. ILC2s express receptors for various humoral factors and thus can change their functions or distribution depending on the environment and circumstances. In this review, we will outline our recent understanding of ILC2 biology and discuss future directions for ILC2 research, particularly in adipose tissue and metabolic homeostasis.

## Introduction

Innate lymphoid cells (ILCs) are predominantly tissue-resident lymphocytes that regulate tissue homeostasis at steady-state. Unlike T and B lymphocytes, ILCs do not express Rag1/2-dependent antigen receptors, but instead express receptors for a variety of humoral factors, including cytokines, metabolites, and neuropeptides. ILCs are classified into three major groups based on their function and developmental pathways; group 1 (natural killer (NK) cells and ILC1s), group 2 (ILC2s), and group 3 (lymphoid tissue inducer (LTi) cells and ILC3s) (1, 2). NK cells circulate in the blood and require the transcription factor eomesodermin (Eomes) for their differentiation, which is important for the expression of cytotoxic molecules such as perforin and granzymes. ILC1s are abundant in the liver and skin, and their differentiation is regulated by the T-box transcription factor (T-bet) but independent of Eomes. Both NK cells and ILC1s are capable of producing interferon (IFN)γ (3). The transcription factor GATA binding protein 3 (GATA3) governs the differentiation of ILC2s. Initially identified in visceral adipose tissue, ILC2s have been found to produce robust amounts of type 2 cytokines such as interleukin (IL)-4, IL-5, and IL-13 (4–6). Subsequent studies have revealed that ILC2s are also present in various other tissues such as lung, skin and gut. Epithelial cell-derived IL-33, IL-25 and thymic stromal lymphopoietin (TSLP) activate ILC2s, with IL-33 being the major activator (7–9). Although ILC2s are normally tissue resident cells, they can migrate from one tissue to another in response to exogenous IL-25 and helminth infection (10). LTi cells are known to produce lymphotoxin (LT), which plays a critical role in the development of lymphoid tissues during fetal stage. ILC3s are mainly found in the adult intestine, and the transcription factor RAR-related orphan receptor γt (RORγt) is critical for their development. ILC3s play an important role in maintaining an optimal intestinal environment by producing the cytokines IL-17A and IL-22 (11).

Adipose tissue is one of the major energy storage sites, consisting of lipid-rich cells called adipocytes as well as stromal vascular fraction comprised of preadipocytes, fibroblasts, vascular endothelial cells, and various immune cells (12). Among immune cells, macrophages are the most abundant (typically 5-10% of stromal cells) and their number increases with obesity (13, 14). Consistently, in obese patients, approximately 40% of stromal cells are macrophages (14). These macrophages ultimately produce large amounts of pro-inflammatory cytokines, inducing chronic inflammation of adipose tissue, leading to insulin resistance, glucose intolerance, and ultimately type 2 diabetes. Furthermore, obesity is thought to increase the risk of severe disease caused by COVID-19 after SARS-CoV-2 infection (15). Therefore, it is an urgent need to establish effective strategies to prevent obesity-related comorbidities. ILC2s were initially discovered in adipose tissue and have been found to mediate type 2 immunity (usually considered as an “anti-inflammatory immune response”). Since then, researchers have begun to explore the key role of ILC2s in the regulation of adipose tissue homeostasis. In this review, we will highlight the recent findings on the interaction between ILC2s and adipose tissue. Furthermore, we aim to discuss the potential of ILC2s as therapeutic targets for metabolic disorders.

## Development of Adipose Tissue-Resident ILC2s

Like other ILCs, peripheral ILC2s emerge during development from common lymphoid progenitors (CLPs) in the bone marrow and fetal liver in a manner dependent on a transcriptional repressor, Id2 (1). Like other lymphocytes such as T cells and B cells, IL-7 and Notch signaling play an important role in differentiation from CLPs. Interestingly, relatively high concentrations of IL-7 and intermediate Notch signaling preferentially induce lineage commitment from CLPs to ILC progenitor cells (16). IL-33 negatively regulates expression of CXC chemokine receptor type 4 on ILC progenitor cells and promotes their exit from the bone marrow (17). Subsequently, ILC progenitor cells migrate to peripheral organs and differentiate into mature ILC2s in a STAT5-dependent manner. At the same time, especially in adipose tissue, platelet-derived growth factor receptor α (PDGFRα)^+^ and glycoprotein 38 (gp38)^+^ mesenchymal cells are thought to further promote ILC2 differentiation, presumably by supplying IL-33. There are also mechanisms that regulate the survival and proliferation of terminally differentiated ILC2s in adipose tissue (18). PDGFRα^+^ multipotent stromal cells in adipose tissue activate ILC2s by producing IL-33. In addition, PDGFRα^+^ multipotent stromal cells directly interact with ILC2s *via* ICAM-1/LFA-1 axis to promote ILC2 proliferation. ILC2s-derived IL-4 and IL-13 stimulate PDGFRα^+^ multipotent stromal cells to produce IL-33 and recruit eosinophils by stimulating eotaxin production. ILC2s also produce IL-5, which further activates eosinophils and maintains a type-2 immune environment in adipose tissue. Collectively, the interaction between ILC2s and PDGFRα^+^ multipotent stromal cells plays a pivotal role in the maintenance of adipose tissue homeostasis.

## Function of Adipose Tissue-Resident ILC2s

As described earlier, ILC2s produce IL-5 and IL-13 in response to IL-33 and recruit eosinophils to adipose tissue (19, 20). Furthermore, ILC2/eosinophil-derived IL-4 and IL-13 induce the differentiation of an anti-inflammatory M2 macrophages and maintain a type 2 immune environment in adipose tissue. On the other hand, numbers of pro-inflammatory M1 macrophages are increased in adipose tissue during obesity. These macrophages produce pro-inflammatory cytokines such as IFNγ, tumor necrosis factor (TNF)α, IL-6, or IL-1β, which significantly dampen the proliferation and function of ILC2s (21). In both mice and humans, the number of adipose tissue-resident ILC2s is markedly reduced in response to obesity (20, 22), and reduced ILC2 function subsequently leads to a decrease in eosinophils and M2 macrophages in adipose tissue, promoting pathological adipogenesis and insulin resistance after high-fat diet (HFD) feeding. On the other hand, adoptive transfer of activated ILC2s into obese mice suppresses HFD-induced weight gain and glucose intolerance (19). ILC2s together with regulatory T cells (Tregs) as well as eosinophils and M2 macrophages, are known to suppress adipose tissue inflammation. Adipose tissue-resident Tregs function as a unique cell population that highly expresses transcription factors GATA3 and peroxisome proliferator-activated receptor γ (PPARγ). IL-33 directly stimulates Treg proliferation, as adipose tissue Tregs highly express the IL-33 receptor ST2 (23, 24). In addition, ILC2s promote Tregs proliferation in adipose tissue through ICOSL and OX40L signaling in response to IL-33 (25).

Adipose tissue is generally categorized into white adipose tissue (WAT), which is found in the abdominal cavity and subcutaneously, and brown adipose tissue (BAT), which is found mainly in the interscapular region in mice and supraclavicular region, neck, para-aorta, paravertebral and suprarenal regions in humans (26, 27). While WAT stores excess energy as triglycerides, BAT produces heat in a way that does not cause shivering in cold conditions, and plays a pivotal role in maintaining body temperature (28). In addition to brown adipocytes, beige adipocytes, a distinct type of cell that regulates thermogenesis *in vivo*, have also been identified (29). Brown and beige adipocytes express uncoupling protein 1 (UCP1), which allows them to decouple mitochondrial respiration from ATP synthesis and dissipate energy as heat. Although brown adipocytes are most abundant in newborns and decrease with age, beige adipocytes can be temporally induced to differentiate within WAT in response to certain stimuli such as cold exposure. This phenomenon is referred to as “WAT browning”. Therefore, beige adipocytes are considered as an attractive target for ameliorating obesity and its related diseases. Two independent groups have reported that ILC2s are involved in WAT browning (22, 30). Artis and colleagues reported that IL-33-activated ILC2s produce methionine-enkephalin peptide (Met-Enk), which activates UCP1 in white adipocytes and promotes beige adipocyte differentiation under cold conditions. On the other hand, Chawla and colleagues showed a slightly different mechanism. Under thermoneutral conditions, ILC2s in WAT recruit eosinophils in adipose tissue in response to IL-33 and produce type 2 cytokines such as IL-4 and IL-13. These cytokines directly activate proliferation of adipocyte precursors expressing IL-4Rα and induce their differentiation into the beige adipocyte lineage. However, the physiological source of IL-33 was not clarified in any of the reports. Since then, studies have demonstrated that adipose-derived stromal cells including mesenchymal cells, mesenchymal stem cells, podoplanin^+^ fibroblasts, or CD31^+^ endothelial cells produce IL-33 at steady state (16, 24, 31–35). A recent study has also implied that eosinophils could be another source of IL-33 in adipose tissues (36). On the other hand, as mice age, mesothelial cells become major producer of IL-33. Therefore, the source of IL-33 might partially vary with age.

ILC2s in adipose tissue significantly decrease with age. Recently, Dixit and colleagues reported that the lethality of the aged mice subjected to cold conditions is considerably higher than that of young mice (37). This may be because ILC2-mediated WAT browning and subsequent heat production are not well induced in aged mice, and thus they are unable to maintain core body temperature. Taken together, these findings suggest that ILC2s play a critical role in maintaining adipose tissue homeostasis.

## Regulation of Adipose Tissues by ILC2s and Signals From Other Organs

Our group has previously demonstrated that ILC2s, which are also present in the small intestine, promote obesity in HFD-fed mice (38). Obesity was induced in HFD-fed *Rag2* ^-/-^ mice (lacking T cells, B cells, and NKT cells but with ILCs or NK cells), but not in *Il2rg* ^-/-^ *Rag2* ^-/-^ mice (lacking all lymphocytes). This result suggests that ILCs are involved in the induction of obesity. As mentioned above, adipose tissue ILC2s (WAT-ILC2s) suppress obesity-induced adipose tissue inflammation. Consistently, we found that adoptive transfer of WAT-ILC2s into *Il2rg* ^-/-^ *Rag2* ^-/-^ mice did not increase the number of M1 macrophages in adipose tissues after HFD-feeding. On the other hand, adoptive transfer of small intestinal ILC2s (SI-ILC2) into *Il2rg* ^-/-^ *Rag2* ^-/-^ mice promoted HFD-induced obesity and subsequent adipose tissue inflammation. Unexpectedly, IL-33 and IL-25 were not involved in SI-ILC2s-dependent obesity induction. In addition to ILC2s, ILC3s (the major ILC population in the intestine) also seem to be partially involved in the process of diet-induced obesity. SI-ILC2s produce higher level of IL-2 than WAT-ILC2s. Interestingly, *Rag2* ^-/-^ mice lacking the β chain of the IL-2 receptor were less sensitive to HFD than *Rag2* ^-/-^ mice, implying that SI-ILC2-derived IL-2 plays an important role in the induction of obesity. Further analysis is required to elucidate the detailed mechanism of obesity promoted by SI-ILC2. Since the microbiota is one of the most important factors affecting lipid metabolism *in vivo*, it will be interesting to investigate the interaction between SI-ILC2s and the microbiota and its potential involvement in the regulation of obesity.

Recently, much attention has been paid to the relationship between ILC2s and the nervous system. The sympathetic nervous system innervating adipose tissue produces catecholamines, which increase the expression of UCP1 in white adipocytes and promote their differentiation into beige adipocytes (39, 40). ILC2s highly express receptors for a variety of neurotransmitters including catecholamines and neuron-ILC2 axis plays a pivotal role in homeostatic control of adipose tissue (41–46). The sympathetic nervous system promotes the production of IL-33 and activates ILC2s in adipose tissue under cold conditions. Conversely, surgical removal or drug-mediated ablation of sympathetic nerves greatly reduces the number of ILC2s and eosinophils in adipose tissue (47). Chronic obesity induces adipose tissue inflammation and severely damages neuronal function (48), resulting in the loss of ILC2s and consequent disruption of adipose tissue homeostasis. Intestinal ILC2s express the β_2-_adrenergic receptors (β_2_-AR) and co-localize with adrenergic neurons. Treatment of mice with β_2_-AR agonist impairs the activation of ILC2s in the intestine (44). In contrast, β_2_-AR signaling does not directly affect the ILC2 function in adipose tissues. Rather, sympathetic nerves stimulate adipose mesenchymal cells *via* adrenergic receptors to accelerate the release of glial-derived neurotrophic factor (GDNF). As a result, GDNF stimulates RET-expressing ILC2s in adipose tissue, promotes the production of IL-5, IL-13, and Met-Enk, and further regulate lipid metabolism (49). ILC2-intrinsic ablation of RET promoted HFD-induced obesity, insulin resistance, and glucose intolerance. These signals from sympathetic nerves are projected from the brain, including the paraventricular nucleus of the hypothalamus, suggesting that the “brain-fat circuit” regulates ILC2 function and adipose tissue homeostasis. In the lung and intestine, ILC2s express the receptor for neuropeptide NMU (41, 45, 46). Thus, NMU can directly activate the ILC2s-mediated type 2 immune response in these tissues. Studies in rats showed that NMU is involved in the regulation of UCP1 expression in adipose tissue, suggesting that the ILC2-NMU axis may also play an important role in adipose tissue (50). Since ILC2s are often located in close proximity to neuropeptide-producing nerves, it is possible that ILC2s produce factors that are beneficial for nerves and also induce proper lipid metabolism. Collectively, inter-organ communication critically influences lipid metabolism, and ILC2s play an important role in this process.

## Discussion

**Figure 1:**
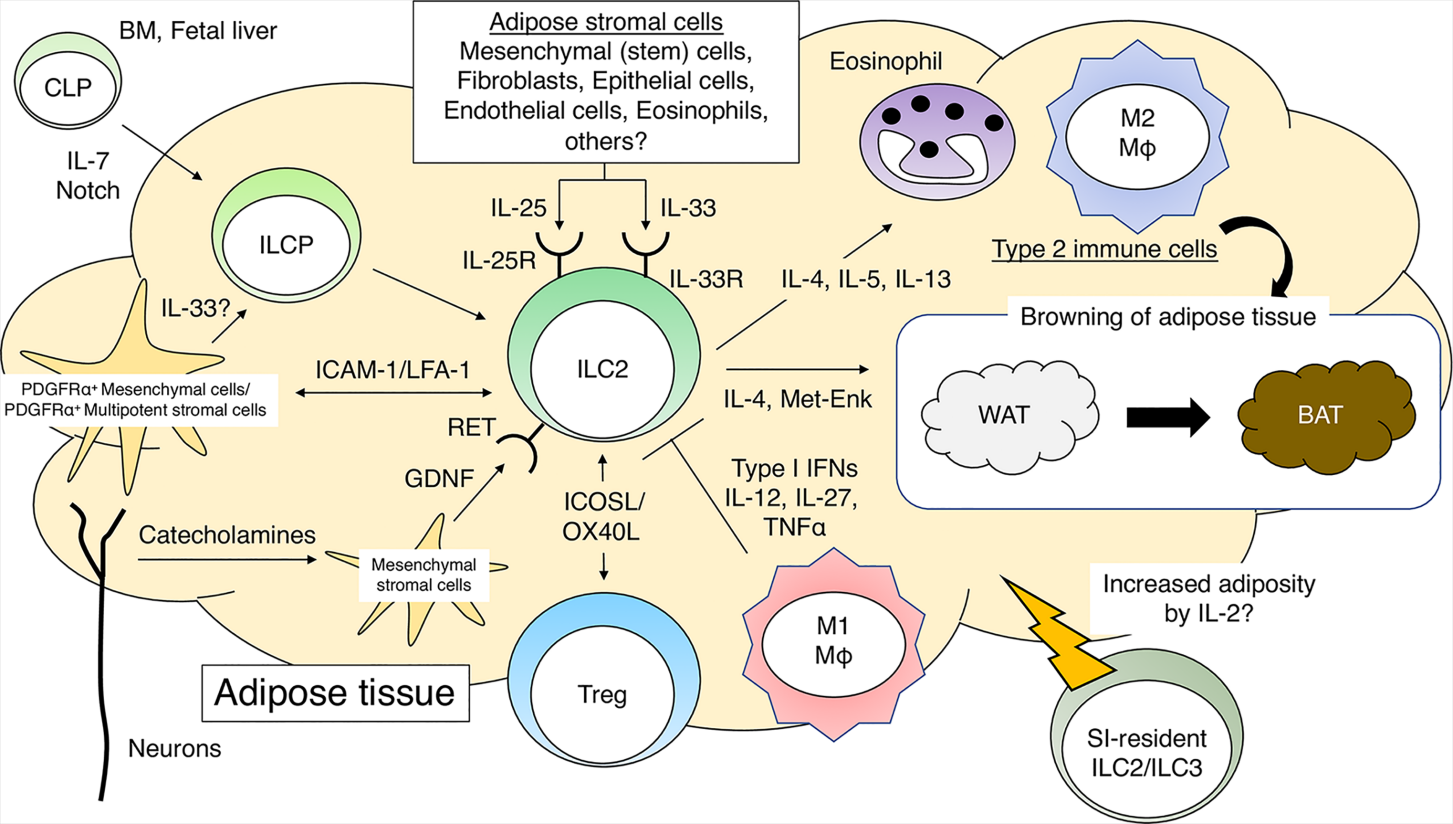
Group 2 innate lymphoid cells (ILC2s) and adipose tissue homeostasis. IL-7 and Notch signaling promote differentiation of common lymphoid progenitors (CLPs) into ILC progenitor cells (ILCPs). PDGFRα^+^gp38^+^ mesenchymal cells further promote the differentiation of ILCP into mature ILC2s in adipose tissues. The direct interaction between ILC2s and PDGFRα^+^ multipotent stromal cells is critical for the maintenance of adipose tissue homeostasis. ILC2s, when activated by cytokines such as IL-25 or IL-33, recruit eosinophils and M2 macrophages to sustain a type 2 immune environment in adipose tissues. ILC2s promote WAT browning by producing type 2 cytokines and Met-Enk. ILC2s promote Treg proliferation in adipose tissues in response to IL-33. Neuron-derived catecholamines activate adipose mesenchymal stromal cells to produce GDNF, which stimulates ILC2s and induces a type 2 immune response. During obesity, M1 macrophages produce pro-inflammatory cytokines and impair the function of ILC2s in adipose tissues. PDGFRα, platelet-derived growth factor receptor α; Met-Enk, Met-enkephalin; GDNF, glial-derived neurotrophic factor.

Here, we have outlined our current knowledge and perspectives on the function of ILC2s in adipose tissue (**Figure 1**). The regulatory mechanisms and functions of adipose tissue differ depending on the site. This is also true for ILC2s. ILC2-mediated adipose tissue browning is observed in subcutaneous WAT, whereas the neuron-ILC2 axis is observed in visceral WAT. Therefore, it should be emphasized that the effects of ILC2s in visceral WAT do not necessarily apply to subcutaneous WAT, and *vice versa*. Nevertheless, there is no doubt that ILC2s play an important role in adipose tissue homeostasis, and therefore, enhancing their homeostatic role may help prevent the development of metabolic abnormalities. Fine-tuning the interactions between ILC2s and adipose tissue components, including both immune and non-immune cells, is also important for maintaining metabolic homeostasis. Identifying the source(s) of cytokines that activate ILC2s, such as IL-33, seems to be one attractive approach.

Obesity reduces the number and function of ILC2s in adipose tissue, and their type 2 immune environment is compromised. In this situation, recruiting ILC2s to adipose tissue would be another attractive approach to prevent the development of adipose tissue inflammation and subsequent metabolic disorders. ILC2s migrate from the intestine to the lung in response to IL-25 and helminth infections, but this phenomenon has not been observed in adipose tissue. Further studies are necessary to fully understand the process by which ILC2s move between tissues to develop methods to recruit ILC2s to adipose tissue. In addition, it should be noted that the overactivation of ILC2s associated with the type 2 immune responses may sometimes be detrimental, as they induce allergic inflammation and intestinal ILC2s promote obesity. ILC2s can change their functions according to the environment and circumstances (51). Therefore, it is essential to study the heterogeneity of ILC2s in different types of adipose tissue in order to find appropriate ways to regulate ILC2-mediated metabolic abnormalities.

## Author Contributions

TM and SK conceived the contents and wrote the manuscript. MW discussed with TM to provide suggestions. All authors contributed to the article and approved the submitted version.

## Funding

This work was supported by a Grant-in-Aid for Scientific Research (B) (21H03388) to TM and a Grant-in-Aid for Scientific Research (A) (20H00511) to SK from the Japan Society for the Promotion of Science, and FRIPRO Mobility Grant Fellowship from the Research Council of Norway (302241) to MW.

## Conflict of Interest

The authors declare that the research was conducted in the absence of any commercial or financial relationships that could be construed as a potential conflict of interest.

## Publisher’s Note

All claims expressed in this article are solely those of the authors and do not necessarily represent those of their affiliated organizations, or those of the publisher, the editors and the reviewers. Any product that may be evaluated in this article, or claim that may be made by its manufacturer, is not guaranteed or endorsed by the publisher.
